# Reduction of in situ force through the meniscus with phased inner resection of medial meniscus: an experimental study in a porcine model

**DOI:** 10.1186/s40634-020-00240-y

**Published:** 2020-04-17

**Authors:** Takehito Hirose, Tatsuo Mae, Yuta Tachibana, Tomoki Ohori, Hiromichi Fujie, Hideki Yoshikawa, Ken Nakata

**Affiliations:** 1 Department of Orthopaedic Surgery, Osaka University Graduate School of Medicine, 2-2, Yamada-oka, Suita-city, Osaka 565-0871 Japan; 2 Department of Sports Medical Biomechanics, Osaka University Graduate School of Medicine, 2-2, Yamada-oka, Suita-city, Osaka 565-0871 Japan; 3 Department of Mechanical Systems Engineering, Tokyo Metropolitan University, 6-6, Asahiga-oka, Hino-city, Tokyo 191-0065 Japan; 4 Department of Medicine for Sports and Performing Arts, Osaka University Graduate School of Medicine, 2-2, Yamada-oka, Suita-city, Osaka 565-0871 Japan

**Keywords:** Medial meniscus, Partial meniscectomy, In situ force, Alignment, 6-degree-of-freedom robotic system

## Abstract

**Purpose:**

Partial meniscectomy can cause osteoarthritic changes in knees, as inner portion as well as peripheral portion of meniscus is important. The hypothesis of this study was that the amount of the inner resection of medial meniscus affected the in situ forces through the meniscus and the tibial varus and external rotation under axial load.

**Methods:**

Fourteen intact porcine knees were investigated with a six-degree of freedom robotic system and force/moment, and the three-dimensional path of intact knees were recorded by universal force sensor when an axial load of 300-N was applied at four different flexion angles (30°, 60°, 90°, and 120°). The same examination was performed on three phased inner resections (30%, 60%, and 90% width) of the medial meniscus. Finally, all paths were reproduced after total medial meniscectomy, and in situ forces of the medial meniscus were calculated based on the superposition principle. Changes in tibiofemoral varus/valgus and internal/external rotation alignment during an axial load were also calculated.

**Results:**

In situ forces of the medial meniscus decreased according to the amount of meniscal resection at all flexion angles. The reduction was significant in knees with inner resections of > 60% width at all flexion angles and even of 30% width at a flexion angle of 120° (*p* < .05). Incremental changes in the tibiofemoral varus alignment increased depending on the inner resection width at all flexion angles (*p* < .05).

**Conclusion:**

The amount of inner resection of the medial meniscus was related to reduction of its in situ forces and increment of the tibial varus rotation under axial load.

## Background

The meniscus plays an important role in load distribution, load absorption/transmission, lubrication, and knee stabilization. Recently, meniscal repair has been recommended to maintain the meniscal function with favorable outcomes [1–3]. However, meniscectomy cannot be avoided in cases of unrepairable degenerative meniscal tears [4].

Meniscectomy has been reported to be a cause of osteoarthritic change in the knees [5, 6], because it affects the tibiofemoral contact area, pressure, or relationship [7–10]. Previous cadaveric studies revealed that small amount of removal of the inner rim in the medial meniscus had little effect on the medial tibiofemoral joint, while more than 50% width of medial meniscal resection significantly altered tibiofemoral contact mechanics [11, 12]. Thus, the amount of meniscal resection might be related to the deterioration in the medial compartment.

The pressure film sensor has been commonly used in biomechanical studies to assess the effects of meniscectomy on knee joints. It could quantify/visualize the tibiofemoral contact pressure or contact area [11–13], although it cannot evaluate the meniscal functions directly, resulting in underestimation the role of the inner portion of the meniscus [12, 13]. In situ force of the meniscus is a novel parameter to represent the resultant force through an injured meniscus [14, 15]. In situ force measurement of the meniscus can be used to directly estimate the meniscal function such as a load transmission and to more sensitively assess the influence of inner resections of the medial meniscus on the tibiofemoral joint. In addition, the evaluation of change in the tibiofemoral relationship can have meaning under an axial load after a phased inner resection of medial meniscus, because the abnormal tibiofemoral relationship leads to osteoarthritic change [9, 16].

Thus, via this study, we aimed to measure the in situ force of the meniscus and tibiofemoral alignment during axial loads with a phased partial meniscectomy. It was hypothesized that the amount of the inner resection of medial meniscus affected the in situ force through the meniscus and the tibial varus and external rotation under axial load.

## Materials and methods

Fourteen intact fresh-frozen porcine knees were used in this study. The pigs were weight of approximately 100 kg and mean age of 6 months. The specimens with cartilage damages or ligament injuries were excluded. As all knee samples were obtained from the food industry and no animals were killed or sacrificed for this study, the study protocol was reviewed and determined not to require oversight by the institutional review board of Osaka University Hospital. The knees were frozen at − 30 °C and thawed at room temperature for 24 h. The tibias and femurs were cut 150 mm in length from the joint line. All muscles, including the quadriceps muscle, patella, and patellar tendon, were carefully removed from the knee, whereas the cruciate or collateral ligaments and capsule were left intact. Each end of the femur and tibia was potted in cylindrical molds of acrylic resin (Ostron II; GC Corp, Tokyo, Japan). The fibula was cut 50 mm in length from the proximal tibiofibular joint and fixed to the tibia using an acrylic resin to maintain its anatomic position. Then, the femoral and tibial cylinders were fixed to the clamps of the manipulator of a robotic simulator.

### Equipment

**Fig. 1:**
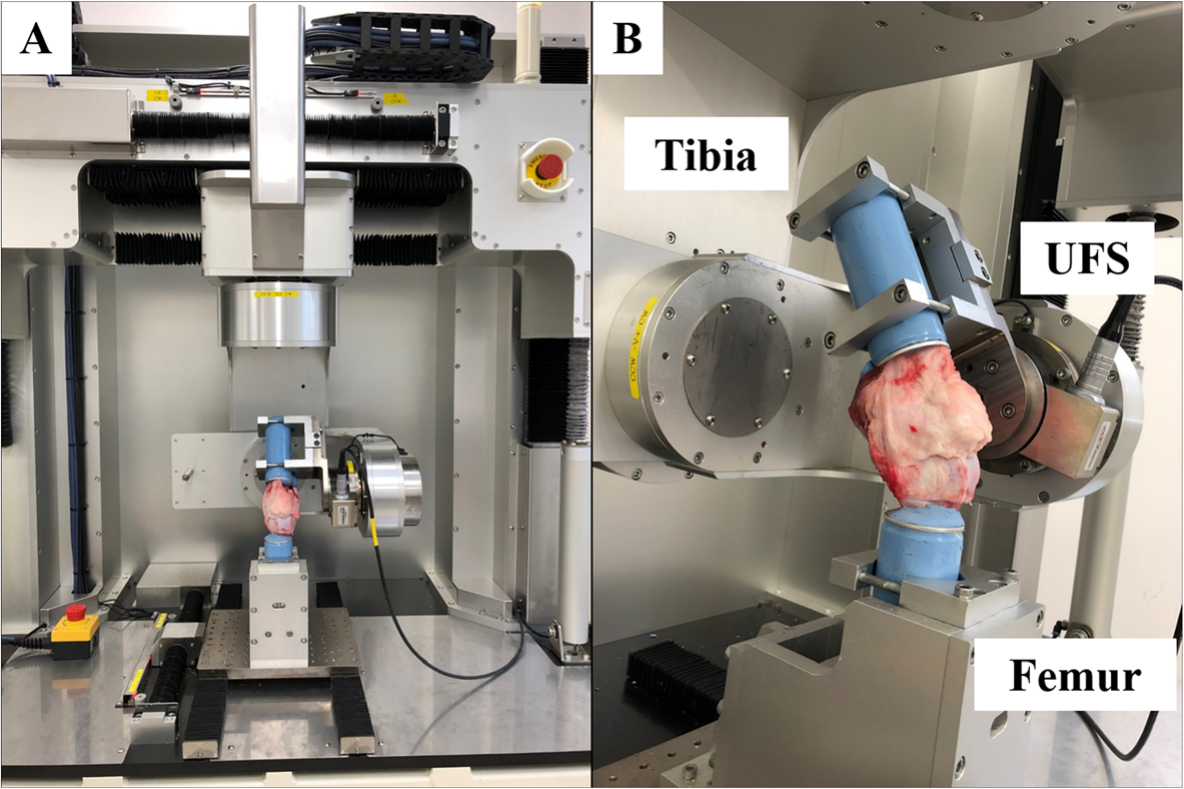
Six-degree of freedom robotic system. **a** A whole image of the robotic system. **b** A knee settled to the simulator via a 6-DOF universal force sensor (UFS)

The robotic simulator system comprised a 6-degree of freedom (DOF) manipulator, servo-motor controllers, and a control computer. The femoral clamp was connected to the lower clamp, and the tibial clamp was fixed to the upper clamp via a 6-DOF universal force/moment sensor (UFS) (IFS-40E, 15A100-I63-EX; JR3, Inc., Woodland, CA, USA) (Fig. 1). The force sensor resolution was 0.01–0.02 N for forces and 0.001 Nm for torques. The test–retest reliability of this robotic system was ±0.006 mm in translation and ± 0.03° in rotation for reproducing recorded paths. Force control fluctuations were < 5 N in force and 0.2 Nm in moment. Thus, this robotic system could apply force/moment to the knee, while controlling the three-dimensional positional displacement and force/moment in the natural joint motion without impeding in any directions [17–19]. The knee joint coordinate system was defined with respect to the non-orthogonal mechanism proposed by Grood and Suntay [20].

### Testing protocol

**Fig. 2:**
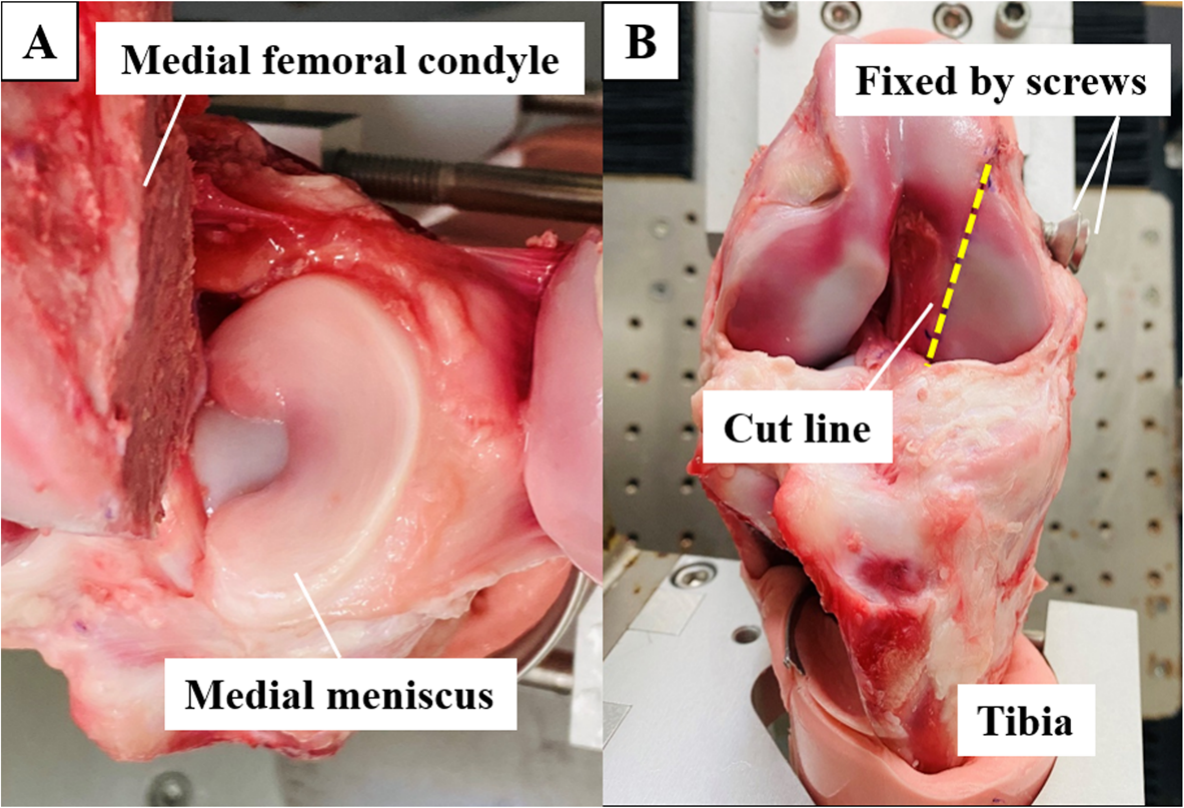
The porcine knee after osteotomy of the medial femoral condyle. **a** Over the top view of medial meniscus. **b** Frontal view of knee after reduction and fixation using two metallic screws

The knee flexion angle was firstly defined as 15° flexion with 1 Nm extension moment because of the intrinsic porcine knee lag. After performing three cycles of passive extension–flexion motion between 15° and 120° of knee flexion under 100-N of axial load for preconditioning, the medial femoral condyle was obliquely osteotomized using a bone saw at 0.50 mm thickness so that the medial compartment including the medial meniscus could be precisely and repeatedly accessed without causing cartilage or soft tissue damage (Fig. 2a) [21]. A pilot study using five samples was conducted to evaluate the effect of the osteotomy on the in situ force of the meniscus as well as the tibiofemoral relationship under a 300-N axial load at 60° of knee flexion, and the osteotomy led to no significant change in these parameters like the previous study [15]. The results were shown in Table 1.

**Table 1:** Differences between pre and post osteotomy in in situ force of medial meniscus and tibiofemoral relationship at 60°of knee flexion (*n* = 5)

	Pre-osteotomy	Post-osteotomy	*p*-value*
In situ force under a 300-N axial load (N)	111.9 ± 10.6	108.4 ± 13.8	0.68
Tibial position (mm) and rotation (°) relative to femur			
Medial(+)/Lateral(-)	2.8 ± 2.2	3.1 ± 3.2	0.46
Varus(+)/Valgus(-)	6.7 ± 2.0	7.0 ± 1.4	0.75
Anterior(+)/Posterior(-)	11.4 ± 7.2	10.4 ± 5.0	0.92
External(+)/Internal(-)	7.4 ± 3.2	9.3 ± 2.2	0.25

Tibial position relative to the femur at 30° of knee flexion was defined as a zero position while suppressing the other force/moment on the knee joint at zero.

*Statistical significance was tested by Mann-Whitney U test

**Fig. 3:**
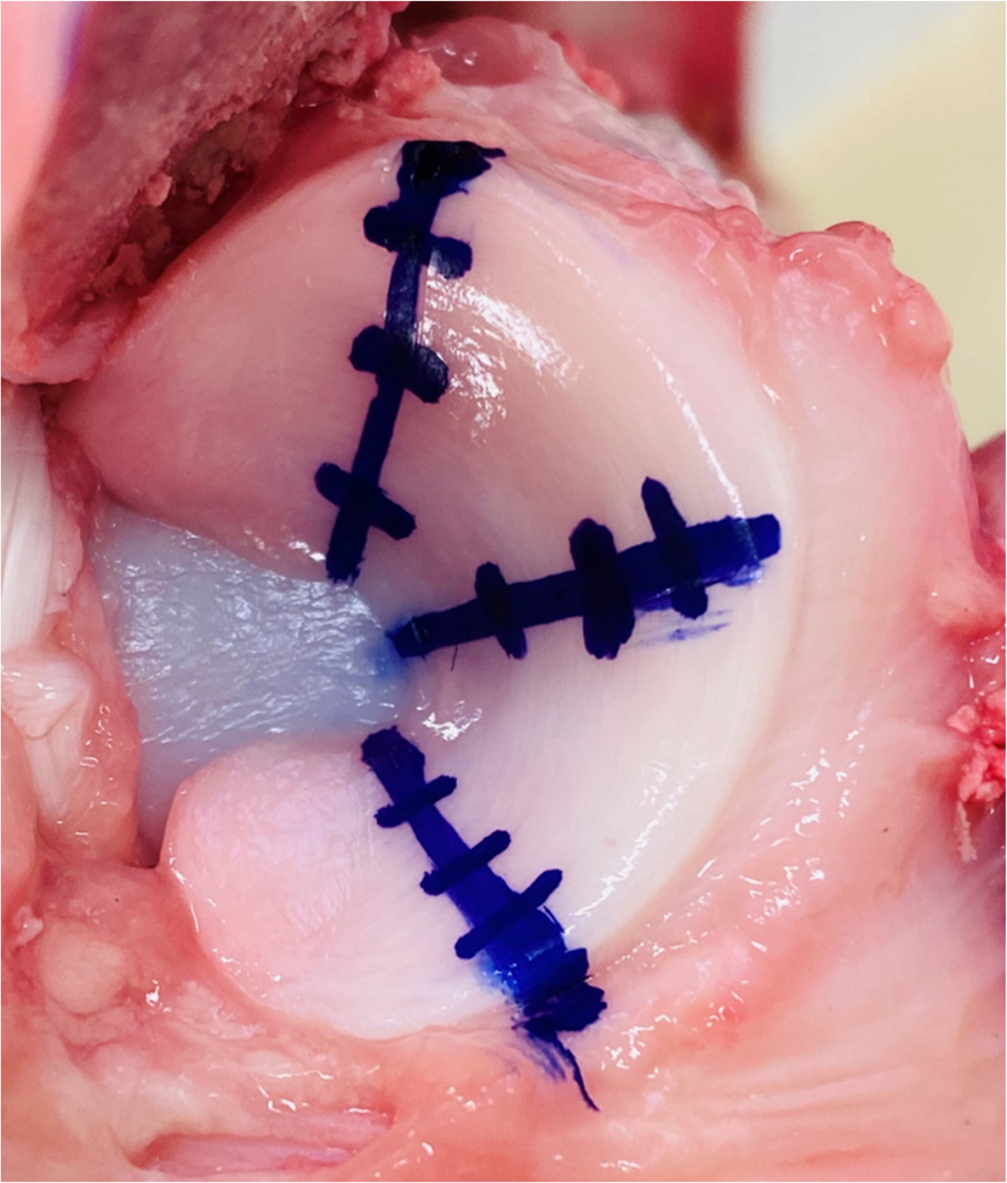
Phased inner resections of the medial meniscus. The inner portion of the meniscus was serially resected (30%/60%/90% width) along a crescent shape of the medial meniscus. Three points (30%, 60% and 90% of width) was marked based on the measurement of total width of meniscus at three segments (anterior, middle and posterior). The anterior and posterior roots were left intact

**Fig. 4:**
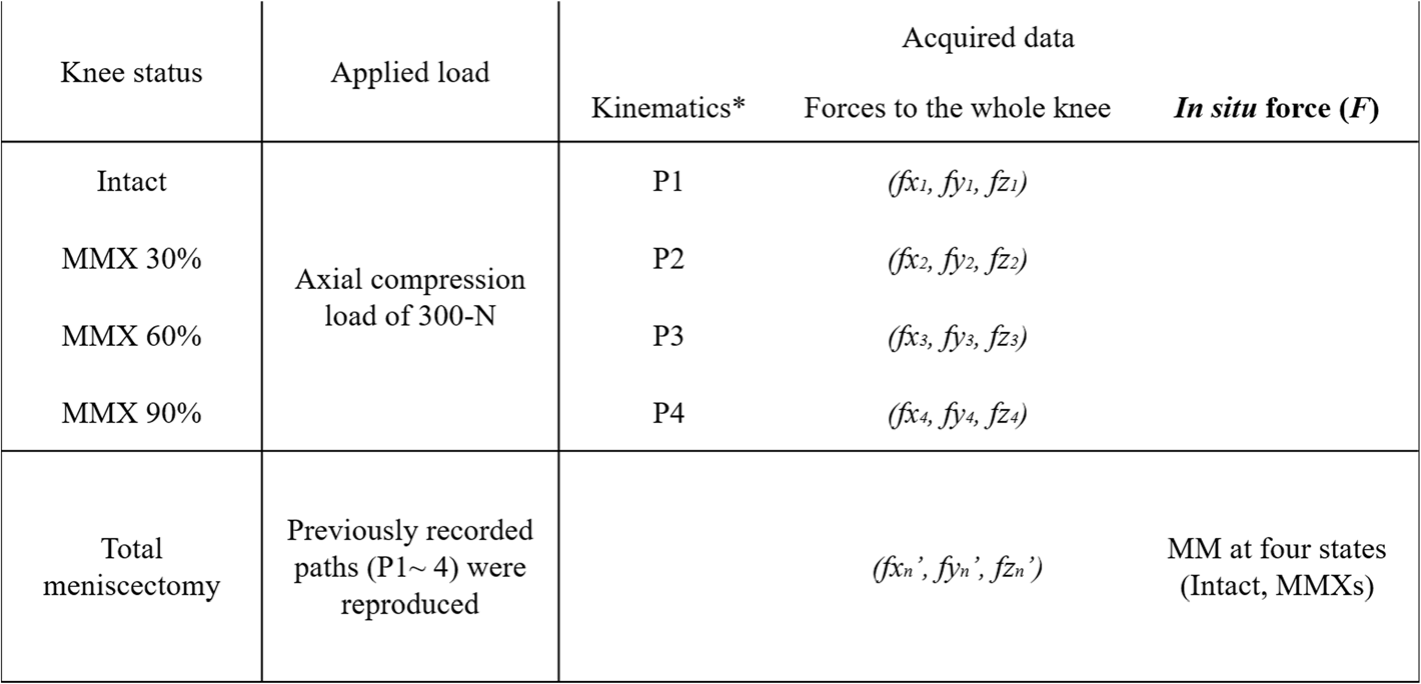
Testing protocol and data acquisition. *Varus/valgus and internal/external changes (°) during compression load

Thus, an “intact knee” was defined as that with the knee status after the medial condyle fixation by two metallic drills of 6.0 mm in diameter in this study (Fig. 2b). Before test, the knee joint was deeply flexed so that the three segments (anterior, middle and posterior) of the medial meniscus could be directly visualized, and three points (30%, 60% and 90% of width) was marked based on the measurement of total width of meniscus with a caliper at three segments (anterior, middle and posterior) (Fig. 3). Subsequently, the simulator applied an axial load of up to 300-N under the force control mode at 30°, 60°, 90°, and 120° knee flexion for three cycles in each flexion angle. The same test protocols were performed after each inner resections (30%, 60%, and 90% width of inner resections) of the medial meniscus (Fig. 3). All three cycles of the three-dimensional paths in the knee motion (P) and the forces of three directions (*fx, fy, fz*) to the knee were recorded using UFS. After performing the test protocols in each meniscal state, the medial meniscus was entirely removed. Then, the simulator reproduced all identical paths previously acquired under the four meniscal states, including the intact and those with inner resections, while forces (*fx′*, *fy′*, and *fz′*) were recorded (Fig. 4).

### Data evaluation

In situ forces of the medial meniscus (*F*) and changes in the tibiofemoral alignment (varus/valgus, internal/external rotation) were evaluated. In situ forces of the meniscus (*F*) under the 300-N axial load was calculated based on the superposition principle [19] using the following formula and as presented in Fig. 4:

$$ F=\sqrt{{\left(f{x}_n-f{x}_n\prime \right)}^2+{\left(f{y}_n-f{y}_n\prime \right)}^2+{\left(f{z}_n-f{z}_n\prime \right)}^{2.}} $$

The results were defined as in situ forces carried only by residual meniscus in response to a load applied to the knee joint, and considered directly correlated with the role of the meniscus in load distribution when axial compressive loads were applied to the knee. Then, in situ forces of the medial meniscus were compared among four different meniscal states (intact, and 30%, 60%, and 90% width of inner resection). Moreover, tibiofemoral varus/valgus changes and internal/external rotation alignment when axial load was applied in each meniscal state (30%, 60%, and 90% width of inner resection) against an intact state were also calculated according to the acquired paths (Pn) using a robotic system. Then, knee alignment changes were compared among the above-mentioned three different meniscal states. For evaluation, data from the third cycle of three testing cycles were used.

### Statistical analysis

Statistical analyses were performed using the JMP software (version 14.3.0; SAS Institute Inc., Cary, NC, USA). Based on a results of initial 4 knees, the required total sample size was nine to detect the difference of 30-N change with a SD of 50-N under the power of 0.80 and 5% level of significance. Thus, the number of knees used in this study was acceptable. After one factor repeated measure analysis of variance to detect the difference among four states of meniscal resection, the Tukey’s honestly significant difference test was performed for the multiple comparisons and the statistical significance was set at a *p*-value of < 0.05.

## Results

**Fig. 5:**
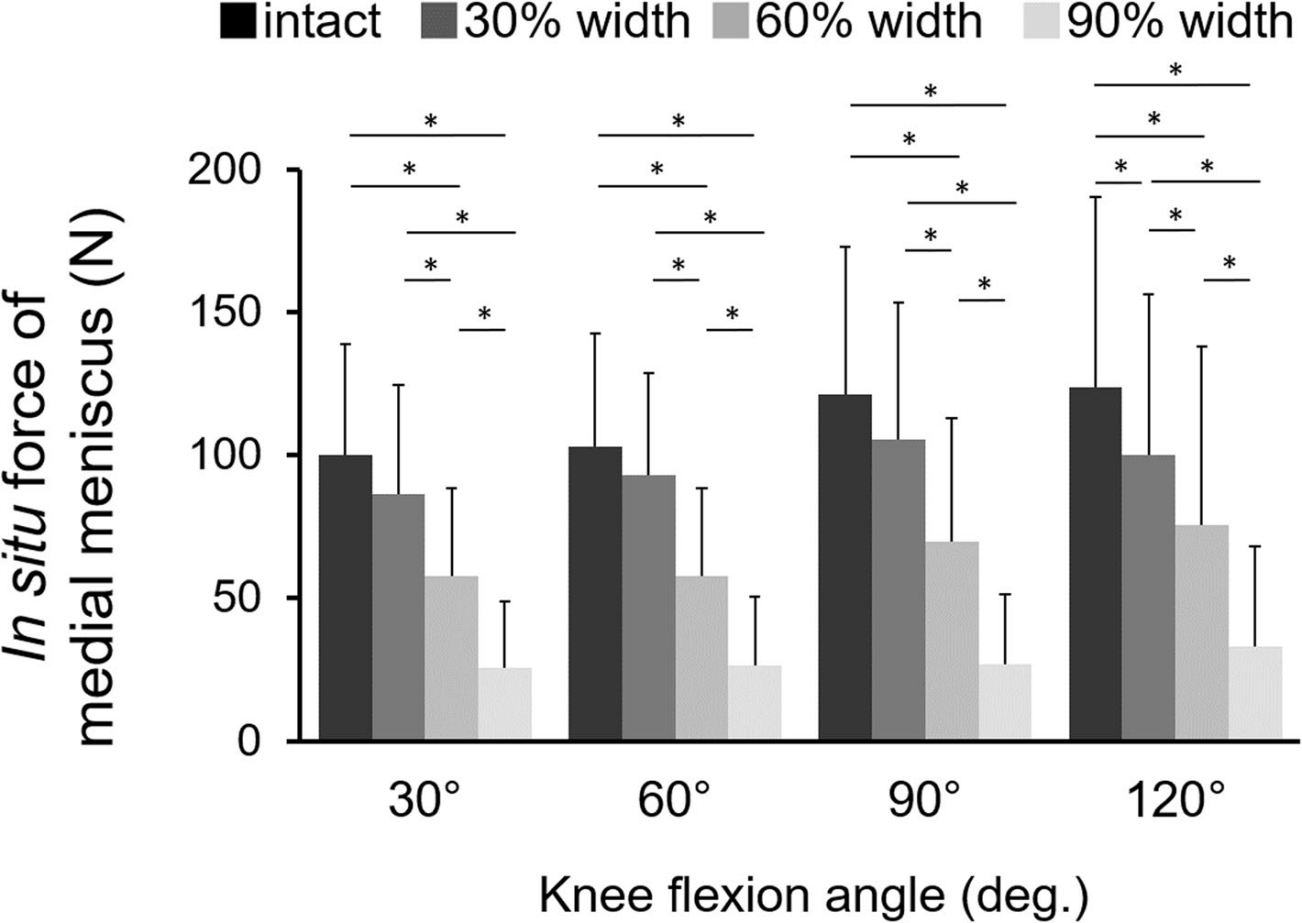
In situ forces of the medial meniscus under an axial load of 300 N. *: significant difference (*p* < .05)

In situ forces of the medial meniscus decreased according to the extent of the inner resection width at all flexion angles, and the in situ force of meniscus with inner resections of 60% and 90% width was significantly smaller than that of intact meniscus at all flexion angles (Fig. 5). Moreover, even the in situ force of meniscus with inner resection of 30% width was significantly smaller than that of intact meniscus at 120° of flexion. No differences were found among different knee flexion angles in each width of the inner resection. The all *p*-values of the statistical comparisons for in situ force of the medial meniscus between each meniscal state were demonstrated in Table 2.

**Table 2:** All p-values of the statistical comparisons for in situ force of the medial meniscus between each meniscal state

	30% width resection	60% width resection	90% width resection
**30°of knee flexion**			
Intact	0.163	< .0001	< .0001
30% width resection	–	0.0004	< .0001
60% width resection	–	–	< .0001
**60°of knee flexion**			
Intact	0.432	< .0001	< .0001
30% width resection	–	< .0001	< .0001
60% width resection	–	–	0.0001
**90°of knee flexion**			
Intact	0.137	< .0001	< .0001
30% width resection	–	< .0001	< .0001
60% width resection	–	–	< .0001
**120°of knee flexion**			
Intact	0.038	< .0001	< .0001
30% width resection	–	0.029	< .0001
60% width resection	–	–	< .0001

**Fig. 6:**
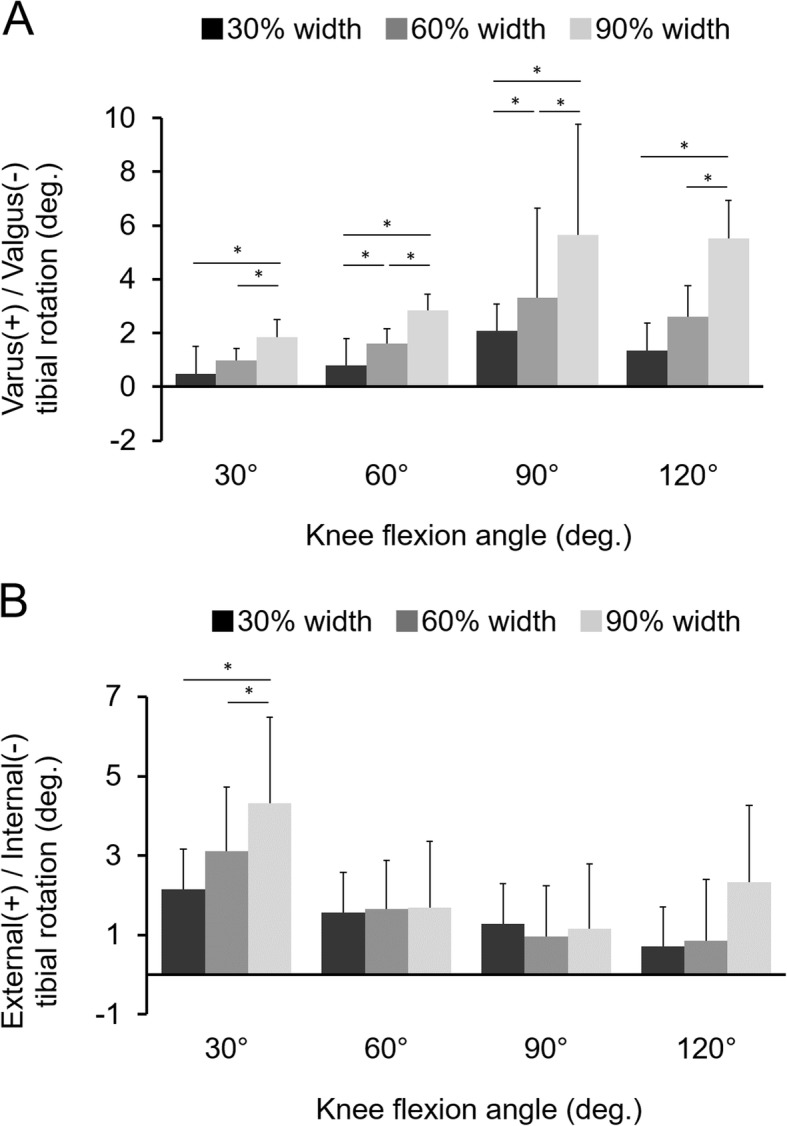
Tibiofemoral relationship under an axial load of 300 N against an intact knee. (**a**) Varus/valgus rotation (**b**) Internal/external rotation *: significant difference (*p* < .05)

Tibiofemoral varus alignment changes under an axial load significantly increased depending on the inner resection width at all flexion angles (Fig. 6a). Conversely, knees consistently displayed external rotation changes under an axial load. Significant difference was only found at a flexion angle of 30° (Fig. 6b). The all *p*-values of the statistical comparisons for tibiofemoral relationships between each meniscal state were demonstrated in Table 3.

**Table 3:** All p-values of the statistical comparisons for tibiofemoral alignment between each meniscal state

	60% width resection	90% width resection
**Varus-valgus**		
**30°of knee flexion**		
30% width resection	0.065	< .0001
60% width resection	–	0.0005
**60°of knee flexion**		
30% width resection	0.001	< .0001
60% width resection	–	0.0002
**90°of knee flexion**		
30% width resection	0.006	< .0001
60% width resection	–	< .0001
**120°of knee flexion**		
30% width resection	0.053	< .0001
60% width resection	–	< .0001
**External-internal**		
**30°of knee flexion**		
30% width resection	0.069	< .0001
60% width resection	–	0.016
**60°of knee flexion**		
30% width resection	0.962	0.933
60% width resection	–	0.996
**90°of knee flexion**		
30% width resection	0.586	0.498
60% width resection	–	0.988
**120°of knee flexion**		
30% width resection	0.977	0.092
60% width resection	–	0.137

## Discussion

The primary finding of this study was that in situ forces of the medial meniscus decreased as the inner portion of the resected meniscus became larger and that inner resection of 90% width reduced the in situ forces of the medial meniscus to approximately 20% of the intact state. In addition, the varus rotational angle under an axial load also increased as the resected inner portion of the meniscus became larger.

Pozzi et al. compared the tibiofemoral contact pressure of intact knees to that with an inner resection of 30% and 75% width of the medial meniscus in canine knees and found that the tibiofemoral contact pressure did not change after an inner resection of 30% width of the medial meniscus but increased after a resection of 75% in width [13]. Freutel et al. assessed the effects of inner resection of the meniscus on the load transmission at the tibial anterior and posterior attachments of the medial meniscus with use of porcine knee as we did. They demonstrated that inner resection over 75% of meniscal width caused an apparent reduction of load transmission, whereas inner resection for 50% width did not show any obvious reduction [22]. In brief, inner rim resection within 50% width might not significantly affect load transmission in previous studies. In these animal studies, care must be taken in interpreting experimental results because the size of the knee differs between studies. However, despite using same size of porcine knees as the previous study used [22], even the inner resection of 30% width decreased in situ force of medial meniscus in our current study. In other words, the inner portion of the medial meniscus was as crucial as peripheral portion in terms of load bearing and it was considered that even a small amount of meniscectomy can result in abnormal load transmission or stress concentration on the cartilage.

In human clinical research, Yoon et al. assessed the effects of arthroscopic medial meniscectomy on knee alignment and showed that the amount of resection was significantly related to varus alignment changes, whereas sex, age, BMI, preoperative alignment, presence of cartilage injury, and follow-up duration were not significantly involved [9]. However, to date, there is no basic information on knee alignment changes during axial compression load after partial meniscectomy. In this in vitro study, the tibiofemoral varus rotation angle under a consistent axial compression load increased according to the width of the inner resection at any flexion angle. Therefore, inner partial meniscectomy brought load axis to the medially and overstress the medial compartment when bearing weight. Recent studies have clarified that varus alignment is related to the medial meniscus extrusion, which also implies the risk of osteoarthritis progression [23, 24]. Therefore, even a small amount of partial meniscectomy might have an effect on tibiofemoral alignment according to the meniscal translation/extrusion.

In our study, the tibia was consistently externally rotated under an axial load despite the meniscal resection width. Because the medial meniscus has been reported to work as a restraint to the tibial rotation [8, 10], the tibia rotated externally after medial meniscectomy. However, the external rotation angle is not related to the amount of resected meniscus in all flexion angles, except for 30°; thus, further investigation might be warranted.

Some limitations of this study should be considered. First, porcine knees were used in this study, whereas a human model might be more clinically applicable. However, this limitation has been overcome because the anatomical structures (including medial meniscal construction) of adult porcine knees are similar to that of adult human knees [25, 26], and several studies have already used porcine models. In addition, young porcine knees were free from degenerative changes; however, most human cadaveric knees are acquired from elderly individuals who might have degenerative changes. Second, the maximum axial load applied to the knees was limited to 300-N because of the robotic system ability, which only resembled to a knee status while standing on its four limbs and did not simulate a more demanding status such as walking or running. If a larger load is applied, in situ forces of the resultant meniscus or the degree of varus alignment changes might increase. Third, the relationship between the in situ meniscus forces and the contact pressure/area in the same specimen was not investigated. The discrepancy between our findings and those of previous studies involving the contact pressure/area should be considered.

## Conclusions

The amount of inner resection of the medial meniscus was related to reduction of its in situ forces and increment of the tibial varus rotation under axial load. The inner rim of the medial meniscus was considered to contribute to load bearing and knee alignment as well as the peripheral portion.
